# Blood transfusion during cardiac surgery dose-dependently stimulates inflammation and coagulopathy in the lung: a case-control study

**DOI:** 10.1186/cc9847

**Published:** 2011-03-11

**Authors:** PR Tuinman, AP Vlaar, AD Cornet, JJ Hofstra, M Levi, JC Meijers, A Beishuizen, MJ Schultz, JB Groeneveld, NP Juffermans

**Affiliations:** 1 Academic Medical Center, Amsterdam, the Netherlands; 2 VU University Medical Center, Amsterdam, the Netherlands

## Introduction

Blood transfusion is associated with increased morbidity and mortality in cardiac surgery patients, but the cause and effect relation remains unknown. We hypothesized that blood transfusion is associated with changes in pulmonary and systemic inflammation and coagulopathy, occurring in patients who do not meet the clinical diagnosis of transfusion-related acute lung injury (TRALI).

## Methods

We performed a case-control study in an ICU of a university hospital. Cardiac surgery patients were grouped as having received no transfusion (*n *= 17), restrictive transfusion (1 to 2 units) (*n *= 18) or multiple transfusions (≥5 units) (*n *= 10). Bronchoalveolar lavage fluid (BALF) and blood were obtained postoperatively. Data are presented as median (IQR).

## Results

Restrictive transfusion increased BALF levels of IL-1β compared with nontransfused controls (*P *< 0.05), and levels were further enhanced by multiple transfusion (*P *< 0.01; 2.9 (9.4) vs. 9.5 (35) vs. 15 (148) pg/ml, respectively). BALF levels of IL-8, TNFα and thrombin-antithrombin complex (TATc) were increased after multiple transfusion compared with nontransfused controls (*P *< 0.01, *P *< 0.001 and *P *< 0.01 respectively), but not after restrictive transfusion. Restrictive transfusion was associated with increased pulmonary levels of plasminogen activator inhibitor-1 compared with nontransfused controls with a further increase after multiple transfusions (*P *< 0.001; 3.4 (1.2) vs. 4.4 (1.4) vs. 6.3 (2.5) ng/ml, respectively). Concomitantly, levels of plasminogen activator activity (PAA%) were lower (*P *< 0.001; 42 (6.5) vs. 34 (6.5) vs. 32 (7.8)%, respectively), indicating impaired fibrinolysis. In the systemic compartment, transfusion was associated with a significant increase in levels of TNFα, TATc and PAA (*P *< 0.05).

## Conclusions

Transfusion during cardiac surgery is associated with activation of inflammation and coagulation in the pulmonary compartment of patients who do not meet TRALI criteria, an effect that was partly dose dependent, suggesting transfusion as mediator of acute lung injury. These pulmonary changes were accompanied by systemic coagulopathy.

